# Prevention of Oxidized Low Density Lipoprotein-Induced Endothelial Cell Injury by DA-PLGA-PEG-cRGD Nanoparticles Combined with Ultrasound

**DOI:** 10.3390/ijms18040815

**Published:** 2017-04-13

**Authors:** Zhaojun Li, Hui Huang, Lili Huang, Lianfang Du, Ying Sun, Yourong Duan

**Affiliations:** 1 Department of Ultrasound, Shanghai First People’s Hospital, School of Medicine, Shanghai Jiao Tong University, Shanghai 200080, China; lzj_1975@sina.com; 2 State Key Laboratory of Oncogenes and Related Genes, Shanghai Cancer Institute, Renji Hospital, School of Medicine, Shanghai Jiao Tong University, Shanghai 200032, China; huanghuiscu@sina.com (H.H.); hll604@126.com (L.H.); yrduan@shsci.org (Y.D.)

**Keywords:** dexamethasone acetate, polymeric nanoparticles, ultrasound, atherosclerosis, polypeptide targeting

## Abstract

In general, atherosclerosis is considered to be a form of chronic inflammation. Dexamethasone has anti-inflammatory effects in atherosclerosis, but it was not considered for long-term administration on account of a poor pharmacokinetic profile and adverse side effects. Nanoparticles in which drugs can be dissolved, encapsulated, entrapped or chemically attached to the particle surface have abilities to incorporate dexamethasone and to be used as controlled or targeted drug delivery system. Long circulatory polymeric nanoparticles present as an assisting approach for controlled and targeted release of the encapsulated drug at the atherosclerotic site. Polymeric nanoparticles combined with ultrasound (US) are widely applied in cancer treatment due to their time applications, low cost, simplicity, and safety. However, there are few studies on atherosclerosis treatment using polymeric nanoparticles combined with US. In this study, targeted dexamethasone acetate (DA)-loaded poly (lactide-glycolide)-polyethylene glycol-cRGD (PLGA-PEG-cRGD) nanoparticles (DA-PLGA-PEG-cRGD NPs) were prepared by the emulsion-evaporation method using cRGD modified PLGA-PEG polymeric materials (PLGA-PEG-cRGD) prepared as the carrier. The average particle size of DA-PLGA-PEG-cRGD NPs was 221.6 ± 0.9 nm. Morphology of the nanoparticles was spherical and uniformly dispersed. In addition, the DA released profiles suggested that ultrasound could promote drug release from the nanocarriers and accelerate the rate of release. In vitro, the cellular uptake process of fluorescein isothiocyanate (FITC)@DA-PLGA-PEG-cRGD NPs combined with US into the damaged human umbilical vein endothelial cells (HUVECs) indicated that US promoted rapid intracellular uptake of FITC@DA- PLGA-PEG-cRGD NPs. The cell viability of DA-PLGA-PEG-cRGD NPs combined with US reached 91.9% ± 0.2%, which demonstrated that DA-PLGA-PEG-cRGD NPs combined with US had a positive therapeutic effect on damaged HUVECs. Overall, DA-PLGA-PEG-cRGD NPs in combination with US may provide a promising drug delivery system to enhance the therapeutic effects of these chemotherapeutics at the cellular level.

## 1. Introduction

According to a recent World Health Organization report, the number of non-communicable diseases (NCDs) in 2012 was 38 million (approximately 68% of 56 million deaths globally), which indicates a serious global public health burden [1,2]. Among NCDs, cardiovascular diseases were the leading cause of death with an estimated 17.5 million deaths in 2012 [3]. Generally, atherosclerosis, which is a disease of the large arteries, primarily leads to heart disease and stroke.

Because of the disease’s etiological complexity, progress involved in determining the cellular and molecular interactions of atherosclerosis has been hindered. With the development of science and technology, a clear understanding of the molecular mechanisms that connect altered cholesterol metabolism and other risk factors to the development of atherosclerotic plaque has been indicated by the new investigative tools. In general, atherosclerosis can be considered to be a form of chronic inflammation resulting from interaction between modified lipoproteins, monocyte-derived macrophages, T cells, and the normal cellular elements of the arterial wall. This inflammatory process can ultimately lead to the development of complex lesions, or plaques, that protrude into the arterial lumen [4,5].

Due to the understanding of the role of inflammation in atherosclerosis, several anti-inflammatory drugs tailored to atherosclerosis are currently under investigation in clinical trials [6,7]. Dexamethasone has anti-inflammatory effects in atherosclerosis, but it was not considered for long-term administration on account of a poor pharmacokinetic profile and adverse side effects [6,8]. Nanoparticles in which drugs can be dissolved, encapsulated, entrapped or chemically attached to the particle surface have abilities to incorporate dexamethasone and be used as a controlled or targeted drug delivery system. Among wide varieties of nanoparticles, long circulatory polymeric nanoparticles present as an assisting approach for controlled and targeted release of the encapsulated drug at the atherosclerotic site [9]. The use of poly (lactide-glycolide) (PLGA) nanoparticles for the encapsulation, delivery, and release of drugs, including dexamethasone acetate (DA), has been well studied in the literature [10]. Platelets targeted glycoprotein Ib (GPIb) conjugated dexamethasone-loaded biodegradable poly (lactide-glycolide) (PLGA) nanoparticles were formulated with the aim to increase the affinity to targeted surfaces, with enhanced controlled release and cellular uptake by activated endothelial cells at the site of vascular injury [11]. Studies have also demonstrated that the surrogate inflammatory marker in atherosclerotic rabbits significantly decreased under the injection of a single intravenous dose of glucocorticoid- encapsulated liposomes conjugated to polyethylene glycol (PEG) [12]. Therefore, we have successfully prepared poly (lactide-glycolide)-polyethylene glycol (PLGA-PEG) nanoparticle- encapsulated dexamethasone acetate (DA) with controlled size and surface characters, which can accumulate at the site of inflammation and angiogenesis through passive targeting. In addition to this approach, site-specific/targeted delivery by the conjugation of specific molecules presents an active area of nanotherapeutics and diagnosis [13]. Targeted delivery requires complimentary ligands of atherosclerotic markers to direct the nanocarriers and to concentrate the therapeutic agent at the target site [14]. It is well known that neovascularization plays an important role in the processes of atherosclerosis. Neovascularization has been correlated with inflammation, which promotes plaque progression, and it may even contribute to plaque rupture as it facilitates cellular trafficking and the recruitment of immune cells through the vasa vasorum [15]. Vascular targeting can be accomplished using nanoparticles that have been functionalized with specific ligands to adhesion molecules such as VCAM1, selectins or integrins such as α_v_β_3_ integrin, as these adhesion molecules are expressed on the activated endothelium of the luminal wall [16] or on the endothelium of newly formed microvessels [17]. Several studies have found that nanoparticles functionalized with specific ligands to α_v_β_3_ integrins can actively target atherosclerotic neovessels [18,19,20,21]. The integrin α_v_β_3_ receptor can recognize and bind a ligand analogue containing the arginine-glycyl-aspartyl (Arg-Gly-Asp, RGD) sequence, so that RGD can act as a targeting molecule for targeting endothelial cells. However, the linear RGD is unstable and easily degraded in vivo. RGD cyclic peptide (cRGD) has a more stable structure [22]. In order to further increase the accumulation at the atherosclerotic site, we have conjugated cRGD, a ligand that binds to α_v_β_3_ expressed in endothelial cells, with PLGA-PEG [22,23,24].

**Figure 1:**
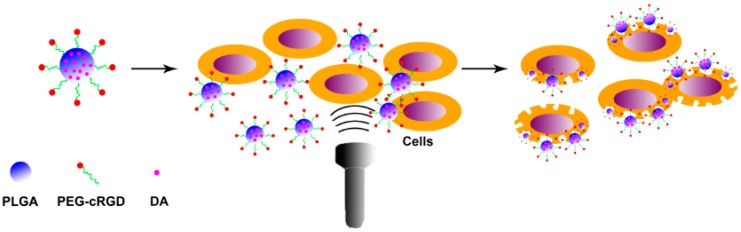
Schematic illustration of the effect of dexamethasone acetate-loaded poly (lactide-glycolide)-polyethylene glycol-cRGD nanoparticles (DA-PLGA-PEG-cRGD NPs) combined with ultrasound (US) on the damaged human umbilical vein endothelial cells (HUVECs).

Ultrasound (US) is widely applied as an imaging modality, resulting from its real time applications, low cost, simplicity, and safety. More recently, studies revealed that US can facilitate local drug and gene delivery, and the encapsulated drug release could be triggered and controlled by US [25,26,27,28,29]. The mechanisms through which US enhances drug and gene delivery can be summarized as direct changes in the drug or vehicle, increasing bioavailability or enhancing efficacy, direct changes in the biological or physiological properties of tissues, facilitating transport, and indirect effects through which US acts on the vehicle to produce changes in the surrounding tissue [30,31,32]. However, there are a few reports of application of US for the treatment of atherosclerosis. In this work (Figure 1), the optimization of nanoparticles combined with US has been determined using an orthogonal design. We then experimentally investigated cellular uptake of the in vitro delivery of US-triggered drug delivery of DA-PLGA-PEG-cRGD nanoparticles (NPs) using confocal microscopy and a flow cytometer. Finally, the therapeutic effect of targeted DA-PLGA-PEG-cRGD NPs combined with US for human umbilical vein endothelial cells (HUVECs) damaged by oxidized low-density lipoprotein (ox-LDL) was evaluated based on cell viability.

## 2. Results and Discussion

### 2.1. Characterization of DA-PLGA-PEG-cRGD Nanoparticles (NPs)

**Figure 2:**
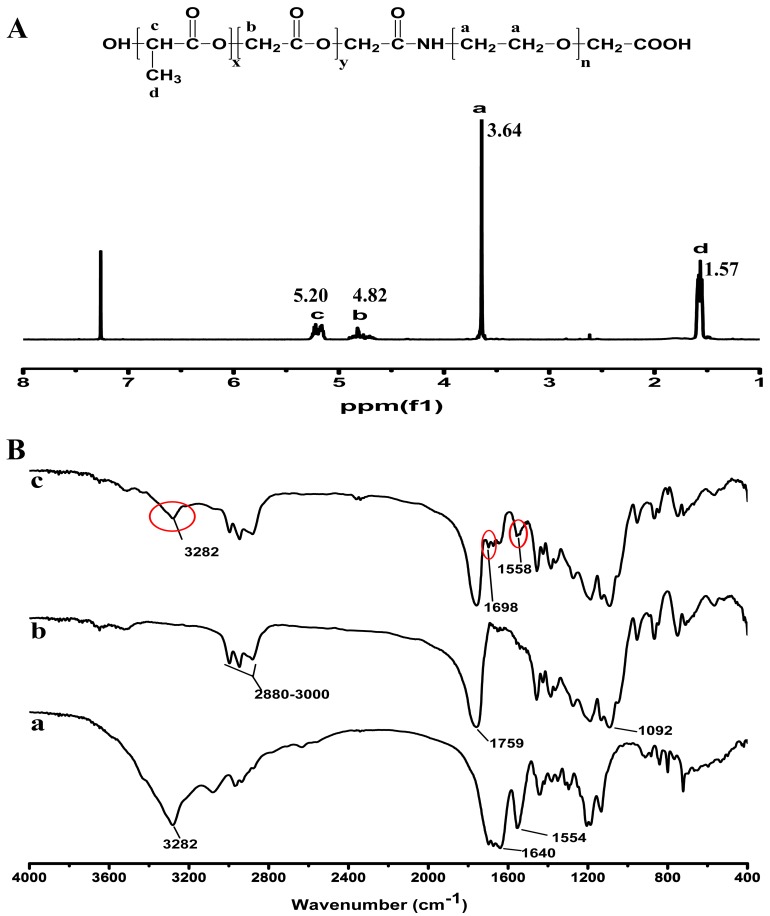
Characterization of the PLGA-PEG-cRGD copolymer. (**A**) ^1^H NMR spectra of PLGA-PEG-COOH in CDCl_3_; (**B**) FTIR spectra of (**a**) cRGD, (**b**) PLGA-PEG-COOH and (**c**) PLGA-PEG-cRGD.

In this study, the PLGA-PEG-cRGD copolymer was synthesized successfully. In the synthesis of PLGA-PEG-cRGD, we used 1-ethyl-3-(3-dimethylaminopropyl) carbodiimide hydrochloride (EDC·HCl) and *N*-hydroxysuccinimide (NHS) as catalysts in order to reduce time. In addition, the carboxyl groups of PLGA reacted with the amine groups of NH_2_-PEG-COOH to form PLGA-PEG-COOH in dichloromethane (DCM) [33]. The crude product of PLGA-PEG-cRGD was dialyzed in distilled water for 24 h to remove EDC, NHS and any residual, non-cross-linked polypeptide. Then, PLGA-PEG-cRGD was obtained by vacuum filtration and dried under vacuum at room temperature. The chemical structure of PLGA-PEG-COOH was confirmed by ^1^H NMR (Figure 2A). The peaks in the ^1^H NMR copolymer spectrum at 1.57 and 5.20 ppm were assigned to the polylactide protons of PLGA, the peaks at 4.82 ppm were assigned to polyglycolide protons and the peak at 3.64 ppm corresponded to the PEG protons [33]. In order to prove the successful conjugation of cRGD to PLGA-PEG, the purified cRGD, PLGA-PEG-COOH and PLGA-PEG-cRGD were evaluated by Fourier transform infrared spectroscopy (FTIR) (Figure 2B). The characteristic FTIR absorption peaks of cRGD were at 3280, 1640 and 1550 cm^−1^, and the above characteristic absorption peaks were observed in the absorption spectra of PLGA-PEG-cRGD.

**Figure 3:**
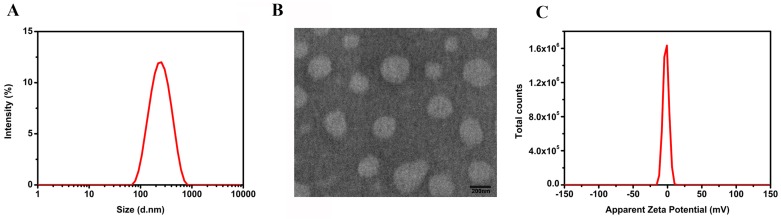
Characterization of the nanoparticles. (**A**) Particle size distribution of DA-PLGA-PEG-cRGD NPs measured by DLS and weighted by intensity; (**B**) DA-PLGA-PEG-cRGD NPs internal structure characterized by TEM. Scale bar, 200 nm; (**C**) Apparent zeta potential of DA-PLGA-PEG-cRGD NPs observed.

The size, polydispersity index (PDI), and zeta potential of the nanoparticles were characterized by dynamic light scattering (DSL). As shown in Figure 3A, the average size of the DA-PLGA-PEG-cRGD NPs was 221.6 ± 0.9 nm, and the PDI was 0.193 ± 0.007, which indicated that the nanoparticles had a narrow size distribution. The DA-PLGA-PEG-cRGD NPs in Phosphate-Buffered Saline (135 mM NaCl, 4.7 mM KCl, 10 mM Na_2_HPO_4_, 2 mM NaH_2_PO_4_, pH = 7.4) were negatively charged with an apparent zeta potential of −2.26 ± 0.08 mV (Figure 3C). Transmission electron microscopy (TEM) was used in further analyses of the particles (Figure 3B). As shown in the TEM image, the nanoparticles were spherical and had no obvious aggregation, and the sizes correlated with the DLS results. The encapsulation efficiency (EE) and loading efficiency (LE) of DA were 89.8% ± 3.7% and 3.5% ± 0.4%, respectively. The results showed that DA-PLGA-PEG-cRGD NPs had an appropriate particle size and good encapsulation efficiency.

### 2.2. Dexamethasone Acetate (DA) Release of the Nanoparticles

**Figure 4:**
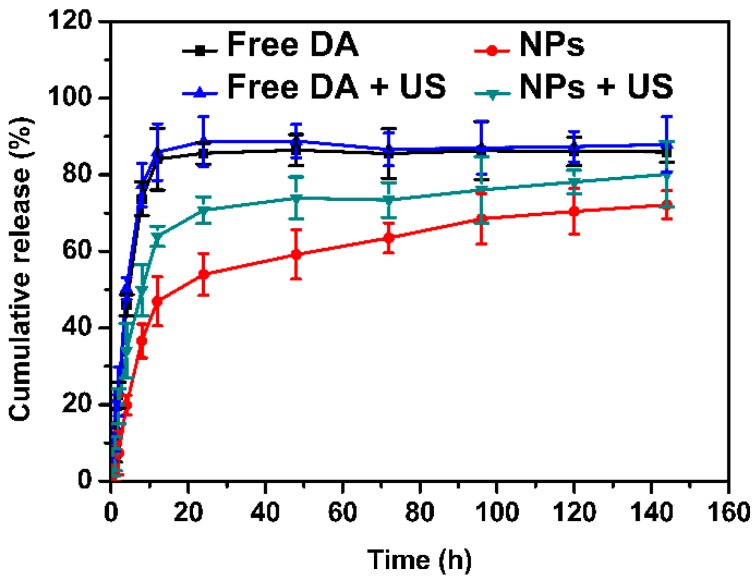
The DA release profiles of DA, DA-PLGA-PEG-cRGD, DA + US, DA-PLGA-PEG-cRGD + US at 37 °C. Data are presented as the means ± S.D. (*n* = 3).

We obtained the in vitro DA release profiles of the nanoparticles by comparing the percentage of the DA released to the amount of DA encapsulated in the nanoparticles. As can be seen from Figure 4, the DA showed a rapid release, and the cumulative amount of drug release reached 83.8% at 12 h. However, the DA was released from the PLGA-PEG-cRGD NPs at a constant rate in two phases. The DA-PLGA-PEG-cRGD NPs displayed an initial burst of drug release of approximately 47.3% at 12 h, followed by a lag-time release phase over a period of 120 h. the cumulative amount of drug released from the DA-PLGA-PEG-cRGD NPs over 144 h was about 71.2%. The results confirmed that the DA-PLGA-PEG-cRGD NPs had a good release property. The initial burst release phase of the DA-PLGA-PEG-cRGD NPs was mainly due to the drug that bound to or adsorbed onto the surface of the nanoparticles. Under a concentration gradient, the drugs that adsorbed onto the surface and near-surface of the nanoparticles desorbed quickly. In addition, the following release phase primarily resulted from the progressive erosion or degradation of the nanoparticle matrix and the drug diffusion through the nanoparticle matrix [34,35].

The DA release profiles of DA + US were similar to those of DA, and the total amount of drug release reached 87.9% at 144 h. When applied with US, the release rates of the DA-PLGA-PEG-cRGD NPs were more rapid than those of DA-PLGA-PEG-cRGD NPs alone, and the cumulative amount of drug released over 144 h was about 80%. The release profiles suggested that ultrasound could promote drug release from the nanocarriers and accelerate the rate of release.

### 2.3. Ox-LDL Induces Vascular Endothelial Cell Oxidative Injury

**Figure 5:**
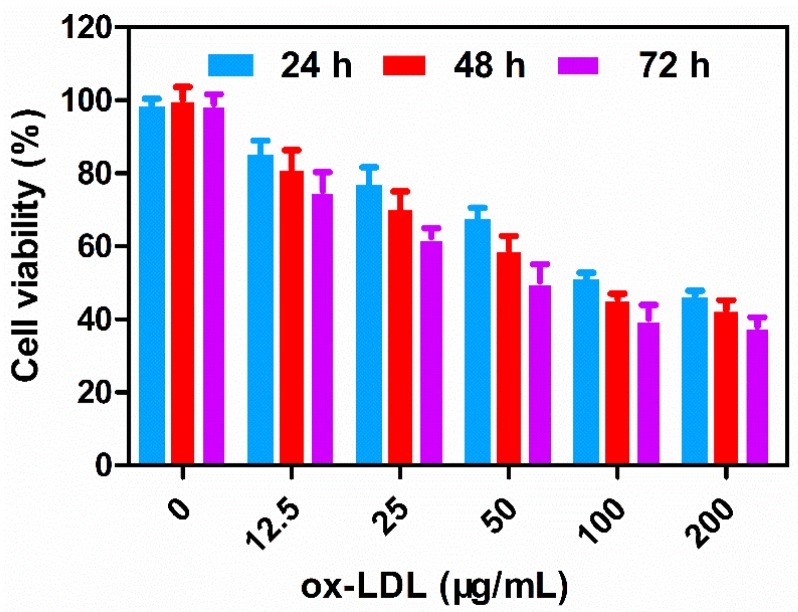
Ox-LDL reduces cell viability of HUVECs in a dose-dependent and time-dependent manner. HUVEC cells were treated with various concentrations of ox-LDL (12.5, 25, 50, 100, 200 µg/mL) for 24, 48, and 72 h. Cell viability was evaluated by the MTT assay. All experiments were repeated at least three times. Data are the mean ± SD. (*n* = 3).

In order to investigate the effect of ox-LDL on HUVEC viability, we performed the MTT (3-(4,5-dimethylthiazol-2-yl)-2,5-diphenyltetrazolium bromide) assay. As shown in Figure 5, the cell vitality of ox-LDL groups was significantly lower than the cell vitality of the control group. The decrease in the viability of HUVECs with increasing concentrations (ox-LDL: 12.5–200 µg/mL) and time (24–72 h) exhibited a dose-dependent and time-dependent effect [36]. As shown in Figure 5, 24 h of exposure to ox-LDL (100 µg/mL, 200 µg/mL) significantly decreased the HUVEC cell viability However, the ox-LDL group (24 h, 200 µg/mL) was seriously damaged; the cell became round and swollen, and the boundary was not clear. Compared with the ox-LDL group (24 h, 200 µg/mL), the cell morphology of the ox-LDL group (24 h, 100 µg/mL) under a light microscope was distorted, while the cell boundary remained clear. Therefore, HUVEC oxidative damage induced by ox-LDL (100 µg/mL) for 24 h was the most suitable condition [36].

Ox-LDL displays a significant toxic effect on vascular endothelial cells [36,37]; the main mechanisms include: (1) ox-LDL has an influence on endothelial progenitor cells (EPC). Studies have shown that ox-LDL can hinder EPC differentiation induced by vascular endothelial growth factors and promote EPC aging, eventually leading to loss of function; (2) ox-LDL induces apoptosis in endothelial cells. As carriers of oxygen free radicals, ox-LDL can directly affect permeability of endothelial cells and regulate the secretion of inflammatory factors, induce endothelial cell apoptosis by some signaling pathways and ultimately cause endothelial dysfunction [38,39,40].

The experimental results showed that ox-LDL could induce HUVEC oxidative damage, and we successfully established a model of oxidative injury by means of this method.

### 2.4. Ultrasound Promotes Rapid Cellular Uptake of DA-PLGA-PEG-cRGD NPs

The nanoparticle concentration and US parameters were selected first in order to obtain the best treatment conditions. After comprehensive analysis, the optimization parameters for the DA-PLGA-PEG-cRGD NPs combined with US were selected as follows: a DA concentration of 20 µg/mL, 30% duty cycle, an ultrasonic time of 90 s, and an ultrasonic intensity of 1.0 W [41].

**Figure 6:**
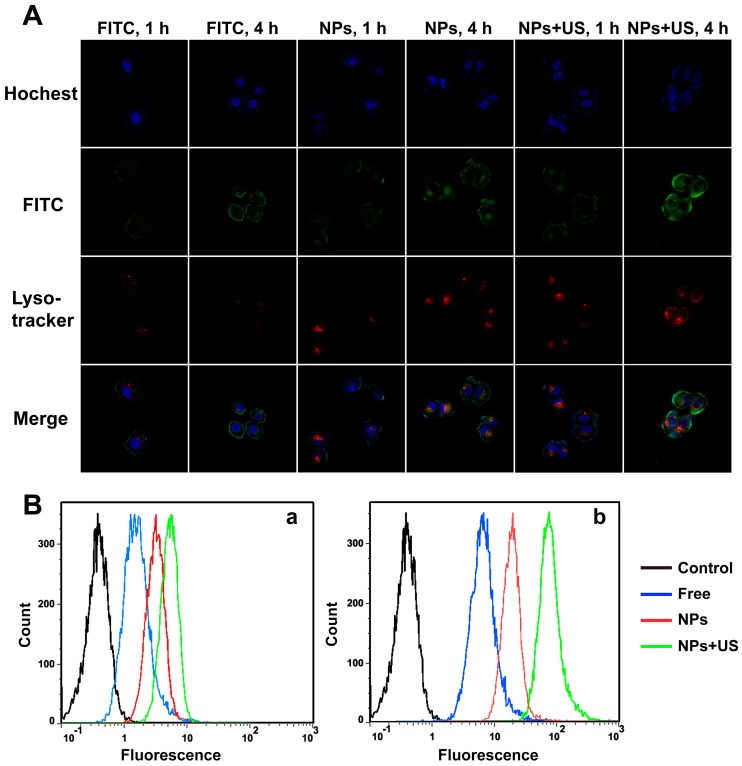
Cellular uptake of the drug in damaged HUVEC cells. (**A**) Confocal microscopy images of intracellular FITC distribution in damaged HUVEC cells after FITC, FITC@DA-PLGA-PEG-cRGD NPs and FITC@DA-PLGA-PEG-cRGD NPs + US treatment for 1 and 4 h. FITC (green) was used as a fluorescent probe; the cell nuclei were stained with Hochest 33342 (blue), and the cell lysosomes were stained with Lyso-Tracker (red). Original magnification, 200×; (**B**) Flow cytometry analyses of damaged HUVEC cells incubated with FITC, FITC@DA-PLGA-PEG-cRGD NPs and FITC@DA-PLGA-PEG-cRGD NPs + US for 1 h (**a**) and 4 h (**b**).

Nanoparticles can efficiently deliver the drug into the cells, which plays an important role in the therapeutic effect [42]. We used confocal microscopy to observe the internalization and intracellular distribution of the nanoparticles in HUVECs damaged by ox-LDL. Fluorescein isothiocyanate (FITC) was used as a fluorescent probe; the cell nuclei were stained with Hocehst 33342, and the cell lysosomes were stained with Lyso-Tracker Red. As shown in Figure 6A, the fluorescent intensity in HUVECs was weak after incubation for 1 h, which suggested that the intracellular drug concentration was not significantly improved in a short time period. However, the fluorescent intensity significantly increased over time. We observed significant fluorescence distributed in the cytosol in damaged HUVECs incubated for 4 h. Furthermore, the fluorescent intensity in HUVECs treated with FITC@DA-PLGA-PEG-cRGD NPs + US was 1.60- and 2.24-fold higher than that of FITC@DA-PLGA-PEG-cRGD NPs and FITC, respectively (Figure S1A). The results showed that US promoted rapid cellular uptake of FITC into damaged HUVECs compared with the cases of FITC and FITC@DA-PLGA-PEG-cRGD NPs [43]. In addition, most FITC@DA-PLGA-PEG-cRGD NPs were taken into cells and distributed in the cytosol in HUVECs after 4 h when treated with FITC@DA-PLGA-PEG-cRGD NPs + US.

In order to further investigate the cellular uptake of FITC@DA-PLGA-PEG-cRGD NPs, we used flow cytometer analysis to analyze the fluorescence intensity of cells incubated under different conditions. According to flow cytometry data (Figure 6B and Figure S1B), the increase in the intracellular fluorescence intensity of damaged HUVECs with increasing time exhibited a time-dependent effect. In addition, after HUVECs were treated with FITC@DA-PLGA-PEG-cRGD NPs + US for 4 h, the intracellular fluorescence intensity was 1.59- and 2.58-fold higher than that of FITC@DA-PLGA- PEG-cRGD NPs and FITC, respectively. The results of the flow cytometer analysis were consistent with the previous cellular uptake results. This also indicated that US promoted rapid intracellular uptake of FITC@DA-PLGA-PEG-cRGD NPs, resulting in much higher cytoplasmic aggregation of DA.

### 2.5. Ultrasound-Improved Therapeutic Effects of DA-PLGA-PEG-cRGD NPs

To evaluate the therapeutic effect of targeted nanoparticles combined with US, we determined the HUVEC viability through an MTT assay. In addition, DA-PLGA-PEG NPs (Figure S2) were prepared for comparison with DA-cRGD-NPs in order to demonstrate the targeting function obtained through the conjugation of cRGD.

**Figure 7:**
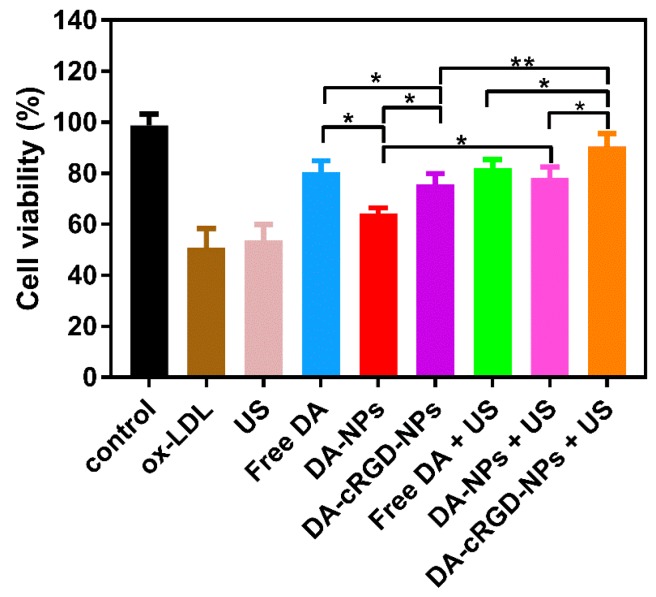
The cell viability of damaged HUVEC cells after treatment with US, DA, DA-PLGA-PEG NPs, DA-PLGA-PEG-cRGD NPs, DA + US, DA-PLGA-PEG NPs + US, DA-PLGA-PEG-cRGD NPs + US. Cell viability was evaluated by the MTT assay. All experiments were repeated at least three times. Data are the mean ± SD. (*n* = 3). ** *p* < 0.001, * *p* < 0.05.

As shown in Figure 7, the therapeutic effects of DA were significantly better than either DA-PLGA-PEG NPs or DA-PLGA-PEG-cRGD NPs. It is possible that the entrapped drug slowly released from the nanocarriers compared with free DA. From previous results, the DA-PLGA-PEG-cRGD NPs displayed an initial burst of drug release, approximately 47.3%, at 12 h, but the DA showed a rapid release, and the cumulative amount of drug release reached 83.8% at the same time. Therefore, the amount of drug from the DA-PLGA-PEG NPs and DA-PLGA-PEG-cRGD NPs taken into the cells decreased, and the therapeutic effects worsened.

There was no significant difference between the free DA group and the free DA combined with US group. However, combined with US, the cell viability of DA-PLGA-PEG NPs and DA-PLGA-PEG-cRGD NPs significantly increased, and the cell viability of DA-PLGA-PEG-cRGD NPs combined with US reached 91.9 ± 0.2%, which demonstrated that DA-PLGA-PEG-cRGD NPs combined with US had a positive therapeutic effect on damaged HUVECs. There may be two reasons, as follows. First, the physical effects generated by ultrasound could promote drug release from the nanocarriers and accelerate the rate of release. Second, US may cause changes in cell morphology, resulting in transient membrane pores, so that the nanoparticles are efficiently taken into cells through transient membrane pores in order to achieve the best therapeutic effect [44,45,46]. As shown in Figure 7, the cell viability of DA-PLGA-PEG-cRGD NPs and DA-PLGA-PEG-cRGD combined with US was higher than that of DA-PLGA-PEG NPs irrespective of combination with US. The results demonstrated that nanoparticles whose PEG chains grafted with cRGD-targeting molecules could effectively target endothelial cells [47].

According to Figure 7, there was no significant difference between the free DA group and the DA-cRGD-NPs combined with US group. Compared with free DA, the therapeutic effects of DA-cRGD-NPs combined with US had a slight improvement at the cellular level. However, in the in vivo study, the concentration of free DA at the target tissue was lower than the administrated concentration after systemic circulation. Furthermore, free DA was not considered for in vitro treatment on account of a poor pharmacokinetic profile and adverse side effects. However, nanoparticles can actively target atherosclerotic neovessels [21] and accumulate at the target site. In addition, the encapsulated drug release could be triggered and controlled by US. Further research should be conducted with respect to the function of ultrasound-assisted nanoparticle drug delivery systems in inflammatory animal models.

## 3. Materials and Methods

### 3.1. Materials

PLGA (*Mn* = 4000–15,000) was purchased from Sigma-Aldrich Co., Ltd. (Shanghai, China). NH_2_-PEG-COOH (*Mn* = 2000) was purchased from Shanghai Yare Biotech (Shanghai, China). EDC·HCl and cRGD (*Mn* = 560) were obtained from GL Biochem Ltd. (Shanghai, China). NHS and sodium dodecyl sulfate (SDS) were purchased from Sigma-Aldrich Co., Ltd. (Shanghai, China). Pluronic F68 was obtained from BASF Co., Ltd. (Shanghai, China), and DA was purchased from Dalian Meilun Biotech Co., Ltd. (Dalian, China). Dimethyl Sulfoxide (DMSO) and DCM were purchased from Aladdin Reagent Co., Ltd. (Shanghai, China). Acetonitrile was obtained from Sinopharm Chemical Reagent Co., Ltd. (Shanghai, China).

Foetal bovine serum (FBS), trypsin, penicillin, streptomycin, and high-glucose Delbecco’s modified Eagle medium (DMEM) were obtained from Biosera. MTT was purchased from Sigma-Aldrich, and ox-LDL was obtained from Dalian Meilun Biotech Co., Ltd. FITC, Hoechst 33342, and Lyso-Tracker Red was purchased from Beyotime Institute of Biotechnology (Shanghai, China).

HUVECs obtained from the Shanghai Cancer Institute (Shanghai, China) were cultured in DMEM containing 10% FBS and 1% antibiotics (100 mg/mL streptomycin and 100 U/mL penicillin) at 37 °C in a humidified atmosphere containing 5% CO_2_.

### 3.2. Methods

#### 3.2.1. Synthesis of PLGA-PEG-COOH

PLGA (150 mg; 1.9 × 10^−5^ mol), EDC·HCl (15 mg; 7.8 × 10^−5^ mol) and NHS (9 mg; 7.8 × 10^−5^ mol) were dissolved in 5 mL of DMSO, and the mixture was stirred for 20 min at room temperature. Then, NH_2_-PEG-COOH (40 mg; 2.0 × 10^−5^ mol) was added to the solution, and the solution was stirred for another 4 h at room temperature. To remove the residual PLGA/NH_2_-PEG-COOH and EDC/NHS, the transparent solution was dialyzed in distilled water for 24 h by using a dialysis bag (MWCO = 3500 Da). Finally, PLGA-PEG-COOH was obtained as a white powder by vacuum filtration and dried under vacuum at room temperature.

#### 3.2.2. Synthesis of PLGA-PEG-cRGD

PLGA-PEG-COOH (100 mg; 1.0 × 10^−5^ mol), EDC·HCl (8 mg; 4.1 × 10^−5^ mol) and NHS (5 mg; 4.3 × 10^−5^ mol) were dissolved in 5 mL of DMSO. After the mixture was stirred for 20 min at 25 °C, cRGD (6 mg; 1.0 × 10^−5^ mol) was added to the solution. Then, the solution was stirred for another 4 h at 25 °C. To remove EDC, NHS and any residual, non-cross-linked polypeptide, the transparent solution was dialyzed in distilled water for 24 h by using a dialysis bag (MWCO = 3500 Da). Finally, PLGA-PEG-cRGD was obtained as a white powder by vacuum filtration and dried under vacuum at room temperature.

The chemical structure of PLGA-PEG-cRGD was confirmed by nuclear magnetic resonance (NMR) spectroscopy. In addition, the ^1^H NMR spectrum was measured in deuterated chloroform using a Bruker Avance 400 (400 MHz) spectrometer (Beijing, China). FTIR was measured using a Nicolet NEXUS-670 Fourier Transform Infrared Spectrometer (Madison, WI, USA).

#### 3.2.3. Preparation of the DA-PLGA-PEG-cRGD NPs

The DA-PLGA-PEG-cRGD NPs were prepared using a conventional emulsification-evaporation method [48]. Briefly, DA (400 µg) was dissolved in DCM and was subsequently mixed with 0.5 mL of DCM containing 10 mg of PLGA-PEG-cRGD. Then, 5 mL of F68 aqueous solution (5 mg/mL) was quickly added to the PLGA-PEG-cRGD/DA solution, and they were sonicated to form an o/w emulsion using a YJ92-II ultrasonic cell pulverizer at 250 W for 2 min. Finally, the emulsion was evaporated to the organic phase under mechanical stirring at room temperature to obtain the DA-PLGA-PEG-cRGD NPs.

#### 3.2.4. Characterization of the DA-PLGA-PEG-cRGD NPs

The particle size, PDI and zeta potential of the DA-PLGA-PEG-cRGD NPs were determined by DLS (Malvern Zetasizer nano ZS, Malvern, UK) measurements. Phosphate-Buffered Saline (135 mM NaCl, 4.7 mM KCl, 10 mM Na_2_HPO_4_, 2 mM NaH_2_PO_4_, pH = 7.4) was used to acquire the particle size, PDI and zeta potential of the DA-PLGA-PEG-cRGD NPs. In order to observe the morphology of the nanoparticles, TEM images were obtained by H-800 transmission electron microscopy (Hitachi, Tokyo, Japan). The procedure for staining was as follows: the nanoparticle solution was first dropped onto a 300-mesh carbon-coated copper grid, and then the excess solution was removed using filter paper. Finally, the grid was allowed to dry at room temperature and was observed using transmission electron microscopy.

#### 3.2.5. Drug Loading

The DA concentration in the DA-PLGA-PEG-cRGD NPs was measured by high performance liquid chromatography (HPLC). According to preliminary experiments, the chromatographic conditions of HPLC were determined: Agilent 1200 HPLC (Shanghai, China), C18 column (Zorbax SB-C18, 250 × 4.6 mm, 5 µm; Agilent), the mobile phase of acetonitrile:water (50:50), flow rate of 1 mL/min, column temperature of 30 °C, detection wavelength of 240 nm, injection volume of 20 µL.

The encapsulation efficiencies (EE) and Loading efficiency (LE) of DA-PLGA-PEG-cRGD NPs were calculated using the formulas:

EE% = (Weight of DA loaded/weight of drug input) × 100%

LE% = (Weight of drug found loaded/weight of drug − loaded nanoparticles) × 100%

#### 3.2.6. Drug Release

In order to assess the DA release profiles in the sound field, an in vitro sonication experiment was performed. DA or DA-PLGA-PEG-cRGD NPs was suspended in 1 mL SDS solution (0.35%) and transferred to dialysis bags (MWCO = 3500 Da) that were placed in a reservoir of 96 mL SDS solution (0.35%) at 37 °C under horizontal shaking (60 rpm/min). The agent was then insonated using the 1 MHz transducer (A Topteam 161 therapeutic ultrasound apparatus, Chattanooga Company, California). The US parameters were selected as follows: 30% duty cycle, ultrasonic time of 90 s, ultrasonic intensity of 1.0 W. At 1, 12, 24, 48, 72, 96, 120 and 144 h, 100 µL solutions were withdrawn, and the same volume of SDS solution (0.35%) was added to the release medium to reach the original volume. The amount of DA released at each time point was analyzed by HPLC as described above.

#### 3.2.7. Effect of ox-LDL on HUVEC Viability

The viability of HUVECs was determined by the MTT assay. In brief, 5.0 × 10^5^ cells were seeded in each well of a 96-well plate and incubated for 24 h at 37 °C with 5% CO_2_. After incubation, non-adherent cells were removed, and HUVECs were incubated with different concentrations of ox-LDL (12.5, 25, 50, 100, 200 µg/mL) for different times (24, 48, 72 h) at 37 °C with 5% CO_2_. Finally, the cells were incubated with 100 µL MTT (0.5 mg/mL) for 4 h at 37 °C with 5% CO_2_. The DMEM medium was removed, and formazan crystals were subsequently dissolved in 150 µL DMSO. The absorbance of the solution was measured at 490 nm.

#### 3.2.8. US Experimental Facilities

A Topteam 161 therapeutic ultrasound apparatus from Chattanooga Company (Chattanooga, CA, USA), with 1 MHz frequency, 100 Hz pulse repetition frequency and 25 mm^2^ cross-sectional area of the probe, was applied for evaluating DA delivery. The facilities were fixed in a supporter with a hole on the upper surface. The transducer was placed on the upper surface, and then 3–5 cm thick couplants were coated to form a conductive pathway of ultrasound waves. Finally, the culture plate was placed onto the surface of the transducer. The experimental parameters were adjusted in the range of 0.5–3.0 W·cm^−2^ (US power), 10–50% (duty cycle), and 1–420 s (US exposure time).

#### 3.2.9. Confocal Microscopy to Determine Cellular Uptake of DA-PLGA-PEG-cRGD NPs

A laser confocal scanning microscope was used to evaluate the cellular uptake of DA in the HUVECs. Briefly, 1.0 × 10^4^ HUVECs were cultured with 1 mL DMEM containing 10% FBS on 20-mm glass-bottom dishes (NEST) and incubated for 24 h at 37 °C with 5% CO_2_. After incubation, the HUVECs were treated with FITC + ox-LDL, FITC@DA-PLGA-PEG-cRGD NPs + ox-LDL, FITC@DA-PLGA-PEG-cRGD NPs + ox-LDL + US containing 20 µg/mL of FITC and 100 µg/mL ox-LDL for different times and were then rinsed three times with PBS. The cells were stained with Lyso-Tracker Red for 30 min at 37 °C with 5% CO_2_ and were washed 3 times with PBS. Subsequently, HUVECs were stained with Hoechst 33342 for 5 min and were washed 3 times with PBS again. Finally, the cells were fixed with 4% paraformaldehyde solution for 30 min at room temperature and were then examined using a confocal laser scanning microscope from the Olympus Optical Company, Ltd. (Tokyo, Japan) to determine cellular uptake of DA-PLGA-PEG-cRGD NPs.

#### 3.2.10. Flow Cytometer Analysis of DA Delivery Efficiency

HUVECs (1.0 × 10^5^) were seeded in 6-well plates with DMEM containing 10% FBS and incubated for 24 h at 37 °C with 5% CO_2._ After incubation, the HUVECs were treated with FITC + ox-LDL, FITC@DA-PLGA-PEG-cRGD NPs + ox-LDL, FITC@DA-PLGA-PEG-cRGD NPs + ox-LDL + US containing 20 µg/mL of FITC and 100 µg/mL ox-LDL for different times. Then, the HUVECs were washed, trypsinized and harvested. The cellular uptake of NPs was measured using a FACScan flow cytometer from Becton Dickinson (Franklin Lakes, NJ, USA).

#### 3.2.11. Therapeutic Effects

The therapeutic effect of nanoparticles combined with US was measured by HUVEC viability, which was determined by the MTT assay. In brief, 5.0 × 10^5^ cells were seeded in each well of a 96-well plate and incubated for 24 h at 37 °C with 5% CO_2_. After incubation, the HUVECs were treated with fresh medium, ox-LDL, free DA + ox-LDL, DA-PLGA-PEG NPs + ox-LDL, DA-PLGA-PEG-cRGD NPs + ox-LDL, free DA + ox-LDL + US, DA-PLGA-PEG NPs + ox-LDL + US, DA-PLGA-PEG-cRGD NPs + ox-LDL + US containing 20 µg/mL of DA and 100 µg/mL ox-LDL for 24 h at 37 °C with 5% CO_2_. Finally, the cells were incubated with 100 µL MTT (0.5 mg/mL) for 4 h at 37 °C with 5% CO_2_. The DMEM medium was removed, and formazan crystals were subsequently dissolved in 150 µL DMSO. The absorbance of the solution was measured at 490 nm.

#### 3.2.12. Statistical Analysis

Statistical analyses of all measurements were carried out using SPSS 17.0 statistical software. All data were expressed as the mean ± standard deviation for each group, and Student’s *t*-test was performed to compare the two groups. A *p*-value < 0.05 was considered statistically signifcant.

## 4. Conclusions

In conclusion, we have developed and characterized targeted DA-loaded PLGA-PEG-cRGD nanoparticles combined with US. DLS analyses of these nanoparticles showed a similar narrow size distribution, and TEM images revealed that the morphology of the nanoparticles was spherical and uniformly dispersed. Furthermore, the DA-released profiles suggested that ultrasound could promote drug release from the nanocarriers. In the in vitro study, HUVECs treated with 100 µg/mL ox-LDL established an appropriate vascular endothelial cell oxidative injury model after 24 h. Confocal microscopy and flow cytometer analysis results indicated that DA-PLGA-PEG-cRGD NPs combined with US had much better delivery efficiency in comparison with free DA or DA-PLGA-PEG-cRGD NPs. As far as therapeutic effects, the cell viability of DA-PLGA-PEG-cRGD NPs combined with US reached 91.9% ± 0.2%, which demonstrated that DA-PLGA-PEG-cRGD NPs combined with US had a positive therapeutic effect on damaged HUVECs. In a word, DA-PLGA-PEG-cRGD NPs in combination with US may provide a promising drug delivery system to enhance the therapeutic effects of these chemotherapeutics at the damaged cell level. However, several factors can influence the therapeutic effects of the in vivo study: the circulation time of this particle, the capacity of drug release in a serum environment, and accessibility to ultrasound treatment. Therefore, the function of the ultrasound-assisted nanoparticle drug delivery system in inflammatory animal models remains to be studied further.

## Supplementary Materials

Supplementary materials can be found at www.mdpi.com/1422-0067/18/4/815/s1.

## Abbreviations

DA	Dexamethasone acetate
PLGA	Poly (lactide-glycolide)
PEG	Polyethylene glycol
DA-PLGA-PEG-cRGD NPs	DA-PLAG-PEG-cRGD nanoparticles
HUVECs	Human umbilical vein endothelial cells
Ox-LDL	Oxidized low-density lipoprotein
US	Ultrasound
FITC	Fluorescein isothiocyanate
NCDs	Non-communicable diseases
EDC·HCl	1-Ethyl-3-(3-dimethylaminopropyl) carbodiimide hydrochloride
NHS	*N*-hydroxysuccinimide
SDS	Sodium dodecyl sulfate
DCM	Dichloromethane
MTT	3-(4,5-dimethylthiazol-2-yl)-2,5-diphenyltetrazolium bromide
PDI	Polydispersity index
DLS	Dynamic light scattering
TEM	Transmission electron microscopy
HPLC	High performance liquid chromatography
EE	Encapsulation efficiencies
LE	Loading efficiency
FTIR	Fourier transform infrared spectroscopy
