# Supplementary material for: Prevention of Oxidized Low Density Lipoprotein-Induced Endothelial Cell Injury by DA-PLGA-PEG-cRGD Nanoparticles Combined with Ultrasound
Source: Int J Mol Sci. 2017 Apr 13;18(4):815. doi: 10.3390/ijms18040815 (PMC5412399; doi:10.3390/ijms18040815)
Supplement: Supplementary file 1 [file ijms-18-00815-s001.pdf]

Supplementary Materials

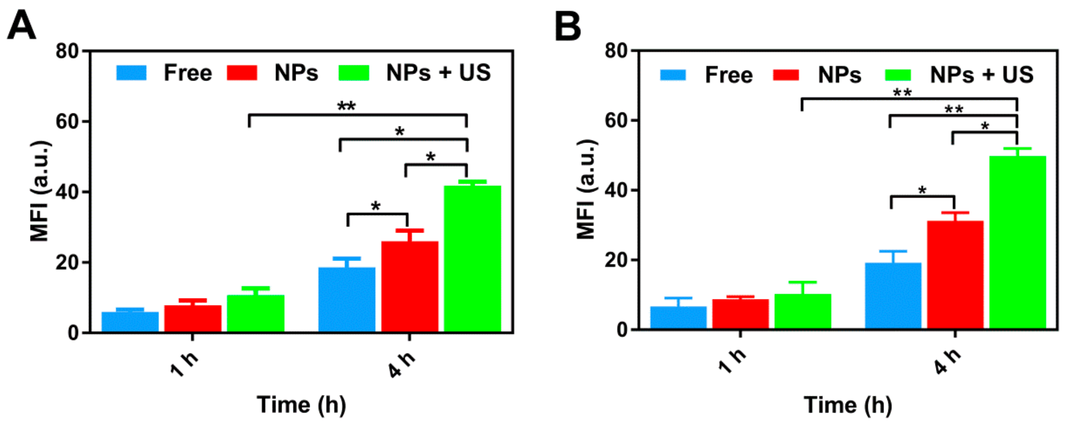

Figure S1. Cellular uptake of drug in damaged HUVEC cells. (**A**) Quantification of confocal microscopy images of intracellular FITC distribution in damaged HUVEC cells after FITC, FITC@DA-PLGA-PEG-cRGD NPs and FITC@DA-PLGA-PEG-cRGD NPs + US treatment for 1 and 4 h; (**B**) Flow cytometry analyses of damaged HUVEC cells incubated with FITC, FITC@DA-PLGA-PEG-cRGD NPs and FITC@DA-PLGA-PEG-cRGD NPs + US for 1 and 4 h. Data shown as mean ± SD. (n = 3). ** $p < 0.001$, * $p < 0.05$.

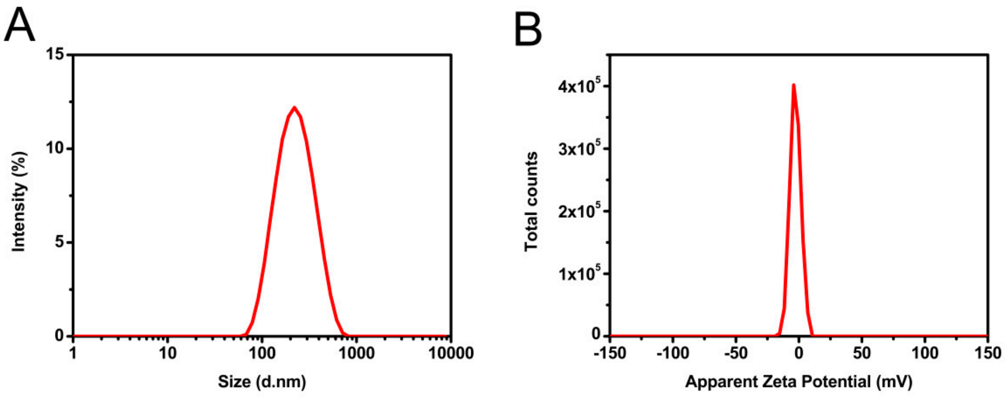

Figure S2. Characterization of DA-PLGA-PEG NPs. (A) Particle size distribution of DA-PLGA-PEG NPs measured by DLS and weighted by intensity. (B) Apparent zeta potential of DA-PLGA-PEG NPs observed.
